# Multiple thermal spin transport performances of graphene nanoribbon heterojuction co-doped with Nitrogen and Boron

**DOI:** 10.1038/s41598-017-04287-3

**Published:** 2017-06-21

**Authors:** Hai Huang, Guoying Gao, Huahua Fu, Anmin Zheng, Fei Zou, Guangqian Ding, Kailun Yao

**Affiliations:** 1 School of Physics and Wuhan National High Magnetic Field Center, Huazhong University of Science and Technology, Wuhan, 430074 China; 2 School of Physics and Optoelectronic Engineering, Yangtze University, Jingzhou, 434023 China

## Abstract

Graphene nanoribbon is a popular material in spintronics owing to its unique electronic properties. Here, we propose a novel spin caloritronics device based on zigzag graphene nanoribbon (ZGNR), which is a heterojunction consisting of a pure single-hydrogen-terminated ZGNR and one doped with nitrogen and boron. Using the density functional theory combined with the non-equilibrium Green’s function, we investigate the thermal spin transport properties of the heterojunction under different magnetic configurations only by a temperature gradient without an external gate or bias voltage. Our results indicate that thermally-induced spin polarized currents can be tuned by switching the magnetic configurations, resulting in a perfect thermal colossal magnetoresistance effect. The heterojunctions with different magnetic configurations exhibit a variety of excellent transport characteristics, including the spin-Seebeck effect, the spin-filtering effect, the temperature switching effect, the negative differential thermal resistance effect and the spin-Seebeck diode feature, which makes the heterojunction a promising candidate for high-efficiently multifunctional spin caloritronic applications.

## Introduction

Spin caloritronics, the combination of thermoelectrics and spintronics, is mainly engaged in research of the relationship between heat transport and spin transport in materials^1–4^. It may solve heat dissipation problems brought by the device miniaturization and thus realize the waste heat recovery, and the control of the spin current induced by a temperature gradient can be used to construct the new type of low-energy-consumption device for information processing^5–7^. Graphene has been closely concerned due to its exceptional properties, including extreme flexibility and stability, high carrier mobility and so on refs 8, 9. Particularly, zigzag graphene nanoribbons (ZGNRs) have attracted considerable interest for potential applications in spintronic devices due to its excellent properties such as spin-filtering effect (SFE), spin-Seebeck effect (SSE), spin-Seebeck diode (SSD), colossal magnetoresistance (CMR), etc^10–12^.

It is well known that the single-hydrogen-terminated ZGNR (ZGNR-H, sp^2^-hybrid) has a magnetic ground state with antiferromagnetic coupling of spin-polarized edge states and the spin-resolved band structures are degenerate^13, 14^, which results in a zero magnetic moment and a non-spin-polarized transport. In order to realize spin-polarized transport through ZGNRs, spin degeneracy should be broken, which can be got by modifying the edge states through various ways, because the magnetism or spin polarized properties in ZGNRs are directly related to the edge states^15–17^. Therefore, the magnetization of ZGNRs can be controlled by using external magnetic fields or through chemical methods, making it show special performance. In experiment, Bai^18^ *et al*. have reported that an extraordinarily large tunable magnetoresistance was achieved by applying a strong perpendicular magnetic field in the field-effect transistor devices based on GNRs. Kim^19^ *et al*. theoretically also predicted very large values of magnetoresistance (MR) in ZGNR-based spin valves by driving the spin-polarized edge states in GNRs from antiferromagnetic (AFM) to ferromagnetic (FM) coupling. Zheng *et al*.^20^ tuned ZGNRs to be either metallic, semiconducting, or even full half-metallic by substitutional doping Nitrogen (N) atoms in one edge and Boron (B) atoms in the other.

**Figure 1:**
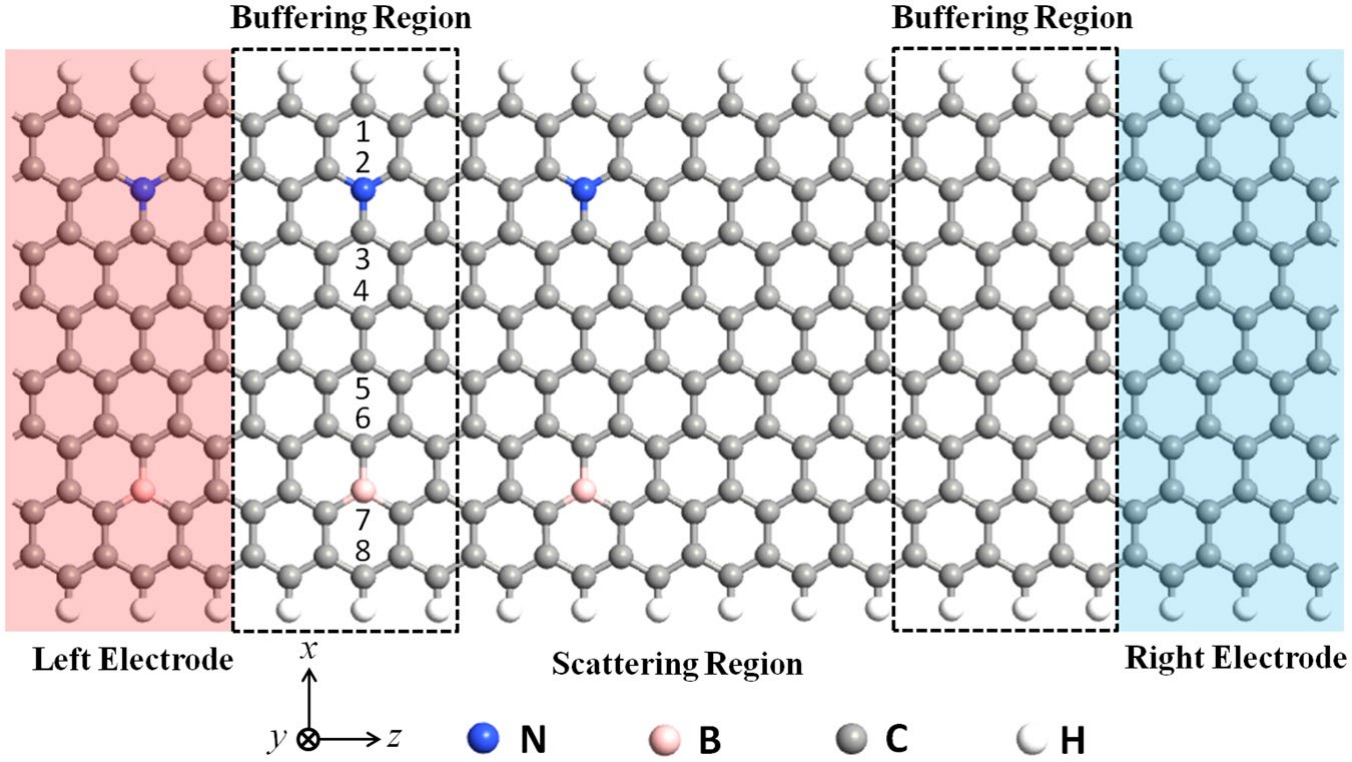
Schematic of the thermal spin device based on the 8-ZGNR-H(N,B)/8-ZGNR-H heterojunction: The device is divided into three regions: the left electrode, the right electrode, and the scattering region. The scattering region also contains three regions: the center heterogeneous region and two buffering regions which are duplications of the left and right electrodes in order to screen the interaction between the electrodes and the center region. A vacuum region of 15 Å is used to eliminate the interactions between adjacent layers (*y* direction). The integers are the ordinal number of zigzag C chains across ZGNR (*x* direction). The right electrode is a pure 8-ZGNR-H and the left electrode is a 8-ZGNR-H doped with N at 2 site and B at 7 site respectively, which are semi-infinite in the transport direction (*z* direction).

Recently, the spin transport and magnetoresistance effects in magnetized ZGNRs and ZGNR-based heterojuctions have been studied^21–23^. However, researchers mostly concerned about the spin currents by a bias voltage, but few about the currents induced by a temperature gradient. Moreover, the comparative study of a ZGNR-based heterojunction under different magnetic configurations is still very lacking^24^. In this work, we design a heterojuction of a 8-ZGNR-H co-doped with N and B (8-ZGNR-H(N,B)) and a pure 8-ZGNR-H (see Fig. 1), and we mainly focus on the spin currents induced by temperature difference between the left and right electrodes. Using first-principles calculation, the spin-resolved electronic structure properties of the electrodes and the transmission spectra under different magnetic configurations are investigated. We are surprised to find that the devices for different magnetic structures show different interesting performances, including SSE, SFE and SSD, etc. Additionally, we also calculate the thermally-induced MR ratios which show multi-values and some are extremely high.

## Results

### Spin-resolved electronic structures

**Figure 2:**
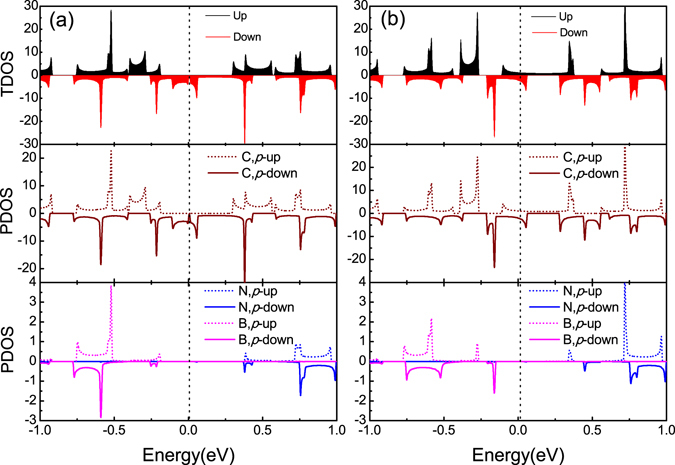
Density of states: The total density of states (TDOS) and projected density of states (PDOS) for the 8-ZGNR-H(N,B) electrode at the AFM state (**a**) and the FM state (**b**). The Fermi level is set to 0 eV.

The choice of electrode will affect the performance of the device, and the general requirement is the band splitting near the Fermi level and the metallicity, in order to realize spin injection and a larger spin flow^25^. Herein, we used single N, B co-doping in the edges to change the electronic structure of 8-ZGNR-H as the left electrode. The total density of states (TDOS) and projected density of states (PDOS) were investigated, as shown in Fig. 2. One can clearly see that 8-ZGNR-H(N,B) at the AFM state is a half-metal^26^ that the spin-down channel exhibits nearly metallicity, whereas the spin-up channel is semiconducting with an energy gap of about 0.49 eV, and 8-ZGNR-H(N,B) at the FM state is a spin-splitting metal, which agree well with previous study^20^. From the PDOS of the p-states of the doped systems, spin splitting occurs in all the atomic orbitals (C, N and B), and the main contribution is the p-state of C, which provides the main magnetic moment.

**Figure 3:**
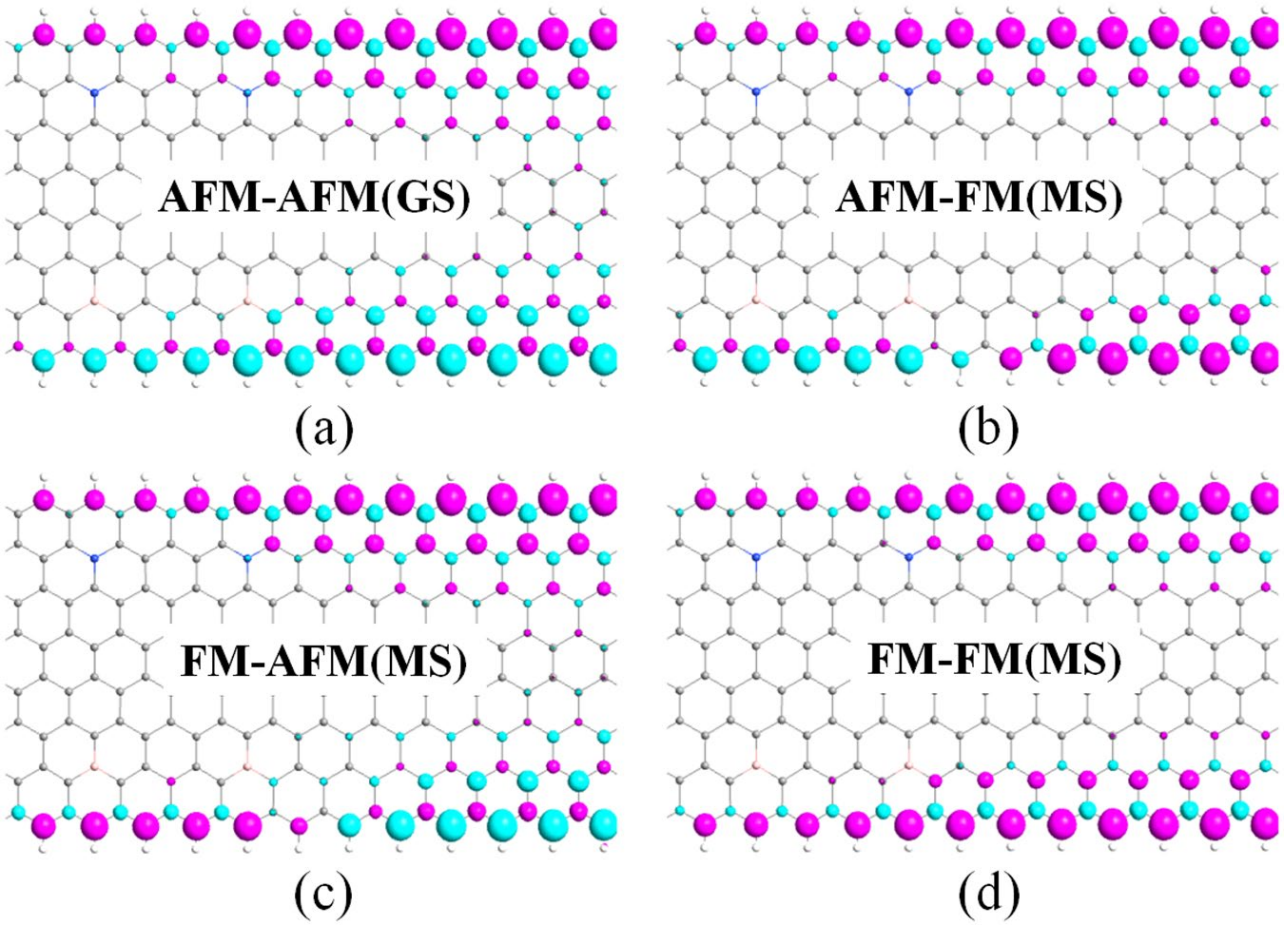
Spin-magnetization density: The isosurfaces of spin density ($\nabla \rho ={\rho }_{\uparrow }-{\rho }_{\downarrow }$) for the scattering region of the heterojunction in four spin configurations: (**a**) [AFM-AFM], (**b**) [AFM-FM], (**c**) [FM-AFM], (**d**) [FM-FM], where ${\rho }_{\uparrow }$ is the spin-up density and ${\rho }_{\downarrow }$ is the spin-down density. [AFM-AFM] is a ground state while [AFM-FM], [FM-AFM] and [FM-FM] are the magnetized states. Phlox and cyan surfaces denote the spin-up and spin-down components, respectively. The isosurfaces value is taken to be ±0.02 e/Å^3^.

The total energies of the 8-ZGNR-H(N,B) electrode at AFM, FM, and nonmagnetic (NM) states were also calculated. The energy differences are Δ*E*_1_ = *E*_AFM_ − *E*_NM_ = −23.5 meV and Δ*E*_2_ = *E*_AFM_ − *E*_FM_ = −1.8 meV, which denotes that the AFM state is the ground state (GS). The large value of Δ*E*_1_ indicates that the NM state is quite unstable, and the small value of Δ*E*_2_ reveals that the AFM and FM states can transform easily each other, which is similar to that of pure 8-ZGNR-H^16, 27^. That is to say, under no external conditions, the 8-ZGNR-H(N,B)/8-ZGNR-H heterojuction we proposed is in the GS state where both the left electrode (8-ZGNR-H(N,B)) and the right electrode (8-ZGNR-H)) are at the AFM states. We can sign this case as the [AFM-AFM] state for the heterojuction, in which the left and right ‘AFM’ represent the spin states of the left and right electrodes, respectively. As we know, ZGNRs can be magnetized by applying an external magnetic field, leading to FM and NM states^19^. Since the NM state is extremely unstable, it will not be considered in this article. So that, when the heterojunction is in a magnetized state (MS), it may have three types of magnetic configurations, i.e., [AFM-FM], [FM-AFM], [FM-FM]. The coupling of the magnetic configurations can be created by means of changing the orientations of local magnetic fields^28^, as shown in Fig. 3. It is obvious that the spin polarization of the heterojunction for the four spin configurations mainly originates from the C atoms at the boundary and rarely from the impurity atoms (N and B), which corresponds to the aforementioned PDOS of the left electrode.

### Thermally-induced spin transport

**Figure 4:**
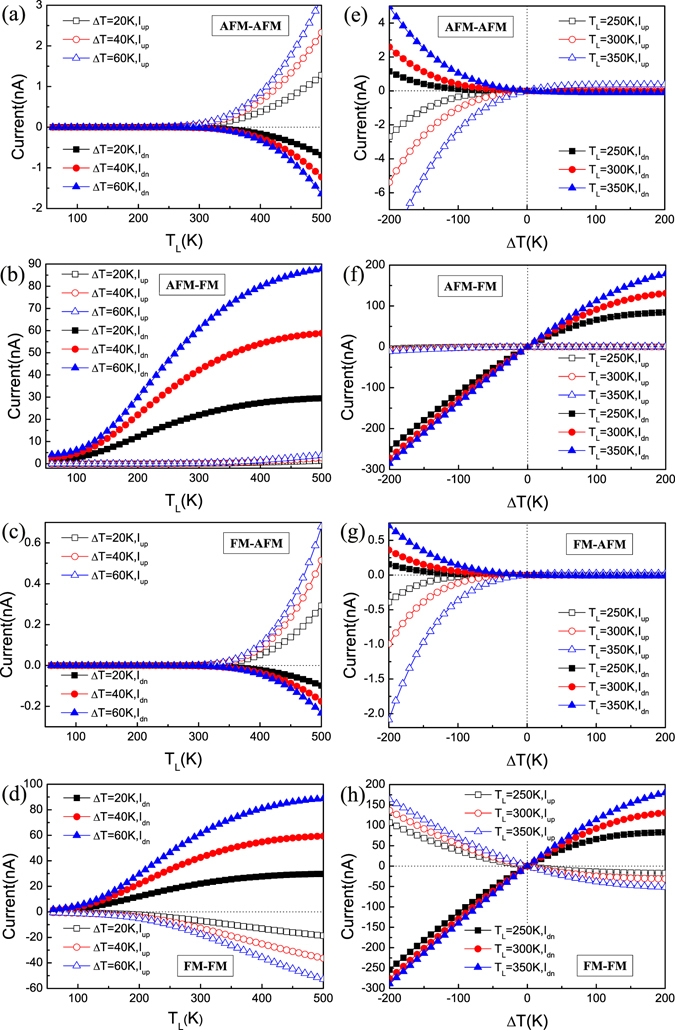
Thermal spin-resolved currents: The thermally-induced spin currents versus *T*_L_ for various Δ*T* (**a**–**d**) and Δ*T* for various *T*_L_ (**e**–**h**) in four spin configurations: [AFM-AFM], [AFM-FM], [FM-AFM], [FM-FM]. Here, *I*_up_ and *I*_dn_ represent the spin-up current and the spin-down current, respectively.

We placed extra emphasis on the thermal spin currents induced by the temperature difference (Δ*T*) without an external gate or bias voltage. Δ*T* = *T*_L_ − *T*_R_, and *T*_L_ and *T*_R_ represent the temperatures of the left and right electrodes, respectively. Figure 4 shows the calculated spin currents of the heterojunction as a function of *T*_L_ (60 K ∼ 500 K) and Δ*T* (−200K ∼ 200 K) in the four spin configurations: [AFM-AFM], [AFM-FM], [FM-AFM], [FM-FM]. It is clear that the spin-dependent currents are generated only by a temperature gradient in all these cases, and the trends for the spin-up current *I*_up_ and the spin-down current *I*_dn_ are similar, that is to say, the spin currents increase with increasing Δ*T* at the same *T*_L_ and with increasing *T*_L_ at the same Δ*T*. However, the current values of [AFM-AFM] and [FM-AFM] are much less than those of [AFM-FM] and [FM-FM] at the same *T*_L_ and Δ*T*. For the [AFM-AFM], [FM-AFM] and [FM-FM] spin configurations, *I*_up_ and *I*_dn_ are of opposite sign, that is, they flow in opposite directions, indicating a SSE. *I*_up_ and *I*_dn_ are of same sign for [AFM-FM], but *I*_up_ is approximately zero in the whole temperature range and *I*_dn_ is extremely large compared with *I*_up_, which shows a perfect SFE (see Fig. 4b,f). From the curves of the spin currents versus *T*_L_ (Fig. 4a–d), we can see that [AFM-AFM] and [FM-AFM] have a threshold temperature which shows a temperature switching effect, but no for [AFM-FM] and [FM-FM]. Through the curves of the spin currents versus Δ*T* (Fig. 4e–h), it is notable that [AFM-AFM] and [FM-AFM] have the SSD feature where both *I*_up_ and *I*_dn_ are asymmetric with respect to zero of Δ*T*. When Δ*T* > 0 (*T*_L_ > *T*_R_), both *I*_up_ and *I*_dn_ nearly equal to zero, while when Δ*T* < 0 (*T*_L_ < *T*_R_), both *I*_up_ and *I*_dn_ increase sharply.

**Figure 5:**
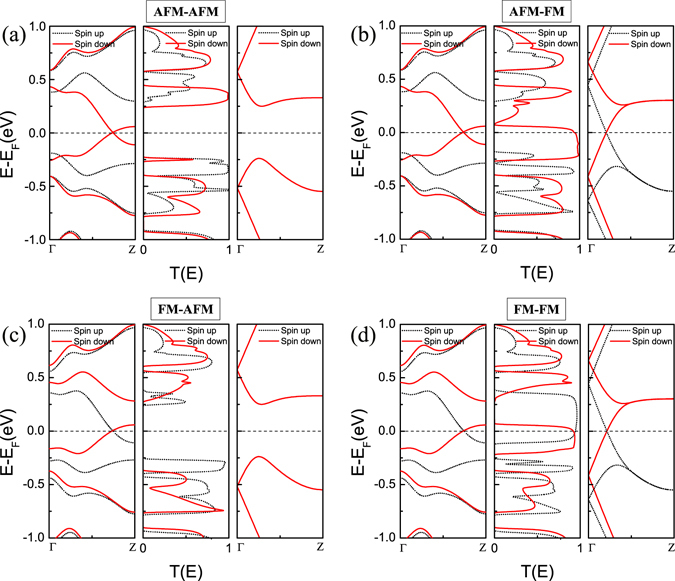
Band structures and transmission spectra: (**a**–**d**) Band structures of the left electrode 8-ZGNR-H(N,B) (left panel) and the right electrode 8-ZGNR-H (right panel), and spin-resolved transmission spectra (middle panel) in four spin configurations: [AFM-AFM], [AFM-FM], [FM-AFM], [FM-FM].

To understand the mechanism of these interesting characteristics mentioned above, it is necessary to analyze the spin-resolved band structures and the transmission spectra of the heterojunction, as shown in Fig. 5. As we know, when the electrodes are at different temperatures, the resultant unbalance in the concentration of thermally-induced charge carriers which is determined by the Fermi distribution (*f*_L_(*E*,*T*_L_) − *f*_R_(*E*,*T*_R_)), allows electrons above the Fermi level *E*_F_ and holes below *E*_F_ to flow from the hot electrode to the cold electrode, resulting in electron current *I*_e_ and hole current *I*_h_, where the direction of *I*_e_ is from the cold electrode to the hot electrode and the opposite direction for *I*_h_. Moving electrons and holes which carry charge and spin can create both charge current and spin current^4^. Considering spin current as research subject, in order to get a net spin current, the spin-resolved transmission spectrum ${{T}}^{\uparrow (\downarrow )}(E)$ must be asymmetric near *E*_F_, that is, the spin-dependent transmittance for the electrons and holes requires to be different, otherwise ${I}_{e}^{\uparrow (\downarrow )}+{I}_{h}^{\uparrow (\downarrow )}=0$ ^29^. As can be seen from the middle panels of Fig. 5a–d, these spin-resolved transmission peaks around *E*_F_ are all distinct and break the electron-hole symmetry, leading to the nonzero net thermal spin currents. Take the case of [AFM-AFM] as an example to further illustrate this point (see the middle panel of Fig. 5a), the transmission peaks for spin-down electrons and holes occur at energies above and below *E*_F_, respectively, but the magnitude and energy scope of the transmission peaks nearly above *E*_F_ are much larger than that below *E*_F_, so *I*_e_ dominates *I*_h_ for the spin-down carriers, resulting in the negative *I*_dn_ (from the right electrode to the left electrode) when Δ*T* > 0 (see Fig. 4a). Meanwhile, the behavior of the spin-up carriers is contrary to that of the spin-down carriers, and the positive *I*_up_ (from the left electrode to the right electrode) is generated, exhibiting a SSE in this spin configuration. A similar effect can be observed in [FM-AFM] (see Fig. 4c) and [FM-FM] (see Fig. 4d), however, *I*_up_ and *I*_dn_ of [FM-FM] are reverse compared with [AFM-AFM] and [FM-AFM], just because *I*_h_ dominates *I*_e_ for the spin-down carriers and *I*_e_ dominates *I*_h_ for the spin-up carriers. Furthermore, in [AFM-FM] (see Fig. 4b), both *I*_up_ and *I*_dn_ are positive due to the fact that *I*_h_ dominates *I*_e_ for both the spin-up and spin-down carriers.

The spin transport performance depends on the specific electronic structure, and the energy band match of the left and right electrodes determines the transport channel open or close in the device^30^. For [AFM-AFM] (Fig. 5a) and [FM-AFM] (Fig. 5c), the right electrode (8-ZGNR-H) at the AFM state is a spin-degenerate semiconductor, which has no band match with the left electrode over a wide energy range near the Fermi level, leading to a transmission gap and a smaller current value compared with [AFM-FM] and [FM-FM]. Moreover, the transmission spectra are relatively far away from the Fermi level for both spin-up and spin-down carriers, and a sufficiently high temperature is required to broaden the curve of the Fermi distribution to overlap with transmission peaks and then turn on the spin currents^31^. Thus a high threshold temperature *T*_th_ (∼250 K for [AFM-AFM] and ∼300 K for [FM-AFM]) is observed. Due to the presence of the band gap for the right electrode in [AFM-AFM] and [FM-AFM], the transport channels open only when Δ*T* < 0 (see Fig. 4e,g), resulting in the SSD feature, and the physical mechanism may be based on the spin-wave excitations in the metal-insulator interface^32^. Inspecting the transmission spectrum of Fig. 5b, we find that there is a remarkably large spin-up transmission gap but no for the spin-down channel, leading to nearly zero spin-up currents over the whole temperature range (see Fig. 4b,f), which shows a SFE in the [AFM-FM] configuration. For [FM-FM] (Fig. 5d), both spin-up and spin-down transmission peaks cross the Fermi level and have no transmission gap, resulting in much larger spin currents without a threshold temperature.

**Figure 6:**
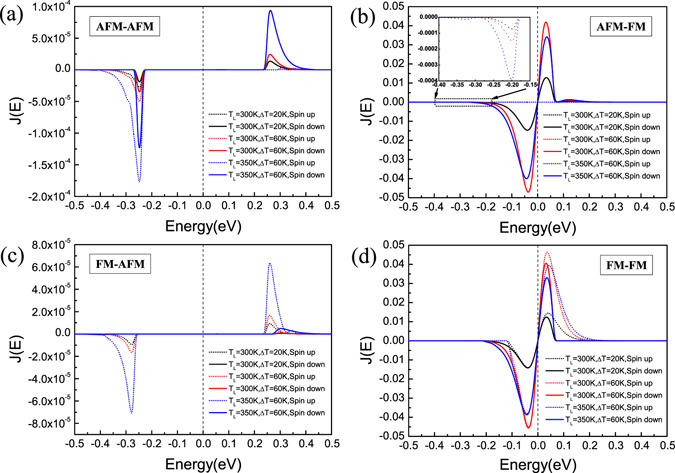
Current spectra: (**a**–**d**) The spin-resolved current spectra with different *T*_L_ and Δ*T* for four spin configurations: [AFM-AFM], [AFM-FM], [FM-AFM], [FM-FM]. The inset is a magnified view for the spin-up current spectra in [AFM-FM].

To quantitatively analyze the spin-resolved current, we calculated the current spectra *J*(*E*) = *T*(*E*)[*f*_L_(*E*,*T*_L_) −*f*_R_(*E*,*T*_R_)], as shown in Fig. 6, where the area enclosed by the curve of *J*(*E*) and the horizontal energy axis reveals the magnitude of current. Taking [AFM-FM] (Fig. 6b) as an example, for the spin-down curves, when *T*_L_ is fixed at 300 K, the peak of the current spectrum at Δ*T* = 60 K is higher than that at Δ*T* = 20 K, indicating that the spin currents increase with increasing Δ*T*. Nevertheless, when Δ*T* is fixed at 60 K, the total area for *J*(*E*) at *T*_L_ = 300 K is smaller than that at *T*_L_ = 350 K, exhibiting that the spin currents increase with the increase of *T*_L_. The spin-up current spectra with quite small values show the same result as the spin-down ones (see the inset of Fig. 6b). Besides, we can also see that *I*_h_ dominates *I*_e_ for both the spin-up and spin-down carriers, because the area below the Fermi level is larger than that above the Fermi level for both the spin-up and spin-down current spectra, which corresponds to the elucidation from the transmission spectra (see Fig. 5b). The tendencies of the current spectra changing with *T*_L_ and Δ*T* for [AFM-AFM], [FM-AFM] and [FM-FM] (Fig. 6a,c,d) are the same as that for [AFM-FM].

### Thermal CMR effect

**Figure 7:**
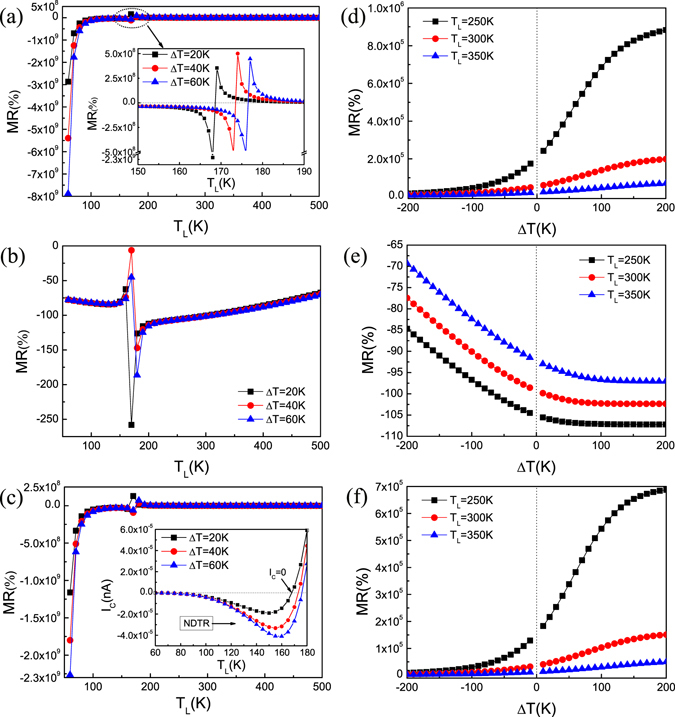
Thermal magnetoresistance: The thermal magnetoresistance (MR) as a function of *T*_L_ with different Δ*T* (**a**–**c**) and Δ*T* with different *T*_L_ (**d**–**f**) by changing the heterojunction from ground state to magnetic states, that is, (**a**) and (**d**) [AFM-AFM] → [AFM-FM], (**b**) and (**e**) [AFM-AFM] → [FM-AFM], (**c**) and (**f**) [AFM-AFM] → [FM-FM]. The inset of (**a**) is a magnified view and the inset of (**c**) is the net electron charge currents *I*_C_ versus *T*_L_ for Δ*T* = 20 K, 40 K and 60 K in the ground state.

The thermal magnetoresistances of the heterojunction switching from the GS to MS states were investigated, which can be obtained from the equation MR(%) = [(*I*_MS_ − *I*_GS_)/*I*_GS_] × 100, where *I*_GS_ and *I*_MS_ are the total electronic charge currents *I*_C_ (=*I*_up_ + *I*_dn_) in the GS state and MS state^33^. Herein, [AFM-AFM] is the GS state while [AFM-FM], [FM-AFM] and [FM-FM] are the MS states, so we can calculate three sets of MR as shown in Fig. 7. The thermal CMR effect is observed, but the MR ratio from [AFM-AFM] to [FM-AFM] (signed as MR_FA_) is much smaller than those from [AFM-AFM] to [AFM-FM] (MR_AF_) and [FM-FM] (MR_FF_) at the same *T*_L_ and Δ*T*. The big difference of the MR ratios essentially originates from the different transmission spectra of the GS and MS states. There is a very wide transmission gap in [AFM-AFM] (GS) (see Fig. 5a), resulting in extremely small *I*_GS_, and similar phenomenon is found in [FM-AFM] (MS) (see Fig. 5c). But [AFM-FM] (MS) and [FM-FM] (MS) have no transmission gap, leading to a very large *I*_MS_, thus MR_AF_ and MR_FF_ are much greater than MR_FA._

As can be seen from Fig. 7a–c, the MR ratios can be tuned by *T*_L_ when Δ*T* is fixed at 20 K, 40 K and 60 K, respectively. Taking MR_AF_ as an example (Fig. 7a), when *T*_L_ < 160 K, the maximum value of MR_AF_ can reach −8 × 10^9^%, and MR_AF_ is negative and larger than 10^7^% in a wide range of temperature from 80 K to 160 K. When *T*_L_ > 180 K, MR_AF_ remains positive with the order of 10^5^, especially near the room-temperature. We are delighted to note that the sign of MR changes from negative to positive over the range of *T*_L_ from 160 K to 180 K at different Δ*T* (see the inset of Fig. 7a), which is called ‘Zigzag’ phenomenon^34^, that is attributed to the reverse of the sign of *I*_GS_ in this temperature range (see the inset of Fig. 7c). Moreover, the ‘Zigzag’ moves to high Δ*T* with *T*_L_ increasing. From the inset of Fig. 7c, one also can see that *I*_GS_ increases to its negative maximum value and then decreases to zero as *T*_L_ increases, which denotes that the negative differential thermal resistance (NDTR) occurs in [AFM-AFM]. The appearance of the NDTR is a consequence of the competition between *I*_up_ and *I*_dn_ with opposite flowing directions, *I*_GS_ = 0 indicates that the thermally-induced pure spin current generates. The sign-reversible CMR effect can also be seen for MR_FF_ (Fig. 7c), which can be widely used in the logic electronics, for example, negative and positive CMR values can be appointed as ‘0’ and ‘1’ signal, respectively. Furthermore, the MR ratios can be tuned by Δ*T* when *T*_L_ is fixed at 250 K, 300 K and 350 K, respectively (Fig. 7d–f). MR increases as Δ*T* increases from −200 K to 200 K at the same *T*_L_ and decreases with *T*_L_ increasing at the same Δ*T*. When Δ*T* < 0, MR_AF_ and MR_FF_ are very small, and when Δ*T* > 0, they can remain at the order of 10^5^, which is a direct manifestation of the SSD effect for [AFM-AFM]. Our findings indicate a perfect CMR effect, which could be applied in thermal spin valve devices.

## Discussion

In summary, using the DFT + NEGF approach, we explored the thermal transport properties of the 8-ZGNR-H(N,B)/8-ZGNR-H heterojuction in different magnetic configurations and elaborated the mechanism for the peculiar properties by analyzing the spin-resolved electronic structures of the electrodes, transmission spectra and the current spectra. Our results indicate that electron transport properties strongly depend on the magnetic configurations, and thermally-induced currents can be controlled by switching the magnetic configurations, leading to a perfect CMR effect which is useful for graphene-based thermal spin valve devices for digital storages and logic operations. The thermally-induced SSE together with SSD feature were observed in [AFM-AFM] and [FM-AFM] with a threshold temperature, showing a temperature switching effect. However, the heterojuction in [FM-FM] has no threshold temperature but generates a larger current value. Moreover, we also found that there is an excellent SFE in [AFM-FM], which can be applied in thermal spin-filtering devices. Overall, the heterojuction we proposed is useful for developing multi-functional spin caloritronic devices.

## Methods

Our calculations have been performed with density functional theory (DFT) combined with non-equilibrium Green’s function technique (NEGF), using the ATK package^35, 36^. The spin-dependent Perdew-Burke-Ernzerhof (PBE) generalized gradient approximation (SGGA) for the exchange-correlation functional and the double-zeta-polarized (DZP) basis set were used for all atoms. The cutoff energy of 150 Ry and the k-points mesh 1 × 1 × 100 were chosen in our work. Structural relaxation was implemented until the force on each atom was less than 0.01 eV/Å and the self-consistency was converged to 10^−5^ eV. The spin-dependent current through the device was obtained by Landauer-Büttiker formula^37^:

1 $${I}^{\uparrow (\downarrow )}=\frac{e}{h}{\int }_{-\infty }^{\infty }{T}^{\uparrow (\downarrow )}(E)[{f}_{{\rm{L}}}(E,{T}_{{\rm{L}}})-{f}_{{\rm{R}}}(E,{T}_{{\rm{R}}})]{\rm{d}}E,$$

where *e* is the electron charge, *h* is the Plank constant, *T*_L(R)_ is the temperature of the left (right) electrode, *f*_L(R)_ is the Fermi-distribution function for the left (right) electrode, and ${T}^{\uparrow (\downarrow )}(E)$ is the spin-resolved transmittance function which can be defined as ref. 38

2 $${T}^{\uparrow (\downarrow )}(E)={\rm{Tr}}{[{{\rm{\Gamma }}}_{{\rm{L}}}{G}^{{\rm{R}}}{{\rm{\Gamma }}}_{{\rm{R}}}{G}^{{\rm{A}}}]}^{\uparrow (\downarrow )}$$

where *G* ^R(A)^ is the retarded (advanced) Green’s functions of the central region and Γ_L(R)_ is the coupling matrix of the left (right) electrode.
